# Recent Advancements in Minimally Invasive Surgery for Early Stage Non-Small Cell Lung Cancer: A Narrative Review

**DOI:** 10.3390/jcm13113354

**Published:** 2024-06-06

**Authors:** Jibran Ahmad Khan, Ibrahem Albalkhi, Sarah Garatli, Marcello Migliore

**Affiliations:** 1 College of Medicine, Alfaisal University, Riyadh 11533, Saudi Arabia; jibrar@alfaisal.edu (J.A.K.); ialbalkhi@alfaisal.edu (I.A.); sarah@alfaisal.edu (S.G.); 2 Thoracic Surgery & Lung Transplant, Lung Health Centre, Organ Transplant Center of Excellence (OTCoE), King Faisal Specialist Hospital & Research Center, Riyadh 12713, Saudi Arabia; 3 Department of Surgery & Medical Specialties, University of Catania, 96100 Catania, Italy

**Keywords:** non-small cell lung cancer, NSCLC, RATS, VATS, lobectomy, segmentectomy, ground-glass opacity

## Abstract

**Introduction:** Lung cancer remains a global health concern, with non-small cell lung cancer (NSCLC) comprising the majority of cases. Early detection of lung cancer has led to an increased number of cases identified in the earlier stages of NSCLC. This required the revaluation of the NSCLC treatment approaches for early stage NSCLC. **Methods:** We conducted a comprehensive search using multiple databases to identify relevant studies on treatment modalities for early stage NSCLC. Inclusion criteria prioritized, but were not limited to, clinical trials and meta-analyses on surgical approaches to early stage NSCLC conducted from 2021 onwards. **Discussion:** Minimally invasive approaches, such as VATS and RATS, along with lung resection techniques, including sublobar resection, have emerged as treatments for early stage NSCLC. Ground-glass opacities (GGOs) have shown prognostic significance, especially when analyzing the consolidation/tumor ratio (CTR). There have also been updates on managing GGOs, including the non-surgical approaches, the extent of lung resection indicated, and the level of lymphadenectomy required. **Conclusions:** The management of early stage NSCLC requires a further assessment of treatment strategies. This includes understanding the required extent of surgical resection, interpreting the significance of GGOs (specifically GGOs with a high CTR), and evaluating the efficacy of alternative therapies. Customized treatment involving surgical and non-surgical interventions is essential for advancing patient care.

## 1. Introduction

Lung cancer is a global health burden that accounts for about 2 million deaths annually [1]. It can be histologically classified into two main types: small cell lung cancer (SCLC) and non-small cell lung cancer (NSCLC). Out of these two, NSCLC accounts for 85% of the cases. NSCLC is an aggressive disease and an early intervention has shown to decrease the 5-year mortality rate [2]. Unfortunately, most cases of NSCLC are detected at advanced stages, with only about 10% of cases identified in stage I [3]. This has a significant impact on the management strategy and prognosis of the disease [4]. It is further highlighted by the fact that the survival rates for stage I, stage II, stage III, and stage IV are 75–90%, 65%, 37%, and 9%, respectively [5,6]. Nevertheless, due to an increase in lung cancer screening, we see a rise in the prevalence of NSCLC in its earlier stages. Early stage NSCLC is typically defined as cancer at stage 2 or lower, providing an opportunity for intervention before the cancer has a chance to further metastasize.

**Figure 1:**
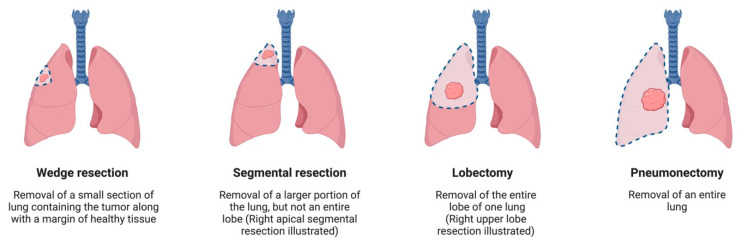
Different techniques of lung resection. Created with BioRender.com.

Most thoracic cancer centers worldwide adopt standard treatment using surgery, radiation, chemotherapy, and palliative care. Surgical treatment plays a key role in managing early stage NSCLC, with an overall survival (OS) of 90% [7]. Surgical approaches for lung cancer may be divided based on the approaches to the surgery and how much lung is excised. Approaches to lung cancer surgery include video-assisted thoracoscopic surgery (VATS), robotic-assisted thoracoscopic surgery (RATS), or traditional open surgery. Resection of the lung is performed as either a wedge resection, segmentectomy, lobectomy, or pneumonectomy (Figure 1). Some specialized centers also utilize emerging treatments such as immunotherapy, targeted therapy, and radiofrequency ablation.

Despite all these treatment modalities, NSCLC exhibits recurrence rates between 30% and 55%, outlining the need for better treatment modalities [8]. Although the recent decade has witnessed multiple advancements in surgical techniques and approaches, unresolved questions remain in the management of early stage NSCLC:

1. How effective are the minimally invasive surgical approaches for NSCLC treatment?
2. Is sublobar resection superior to lobectomy for stage IA NSCLC treatment?
3. What is the prognostic value of ground-glass opacities (GGOs).
4. Is sublobar resection superior to lobectomy for GGOs?
5. What is the extent of the lymphadenectomy required for GGOs?

We aim to include a review of the literature on these four questions to update physicians on the most recent surgical advancements in the management of NSCLC. Additionally, we briefly alluded to recent updates in the non-surgical approaches to NSCLC.

## 2. Methods

To identify all studies evaluating the treatment modalities for early stage NSCLC, we performed a Boolean search using “non-small cell lung cancer”, “NSCLC”, “RATS”, “VATS”, “lobectomy”, “segmentectomy”, “lymphadenectomy”, “lymph node dissection”, “ground-glass opacity”, “targeted therapy”, “immunotherapy”, “chemotherapy”, and “radiotherapy” keywords. Two authors (J.K., I.A.) reviewed and included the relevant full-text articles in the English language using the PubMed/MEDLINE, Web of Science, Scopus, and Google Scholar databases. Afterward, the authors (J.K., I.A.) performed a more comprehensive review of the full manuscripts for inclusion. There was no year limit set. The inclusion criteria were prioritized but were not limited to clinical trials and meta-analyses on the surgical approaches of early stage NSCLC published from 2021 onwards.

## 3. Discussion

### 3.1. Surgical Approaches to NSCLC Treatment

#### 3.1.1. How Effective Are the Minimally Invasive Surgical Approaches for NSCLC Treatment?

In the past, open lobectomy or pneumonectomy with mediastinal lymph node dissection was the primary surgical management approach. Nowadays, minimally invasive thoracoscopic techniques are becoming increasingly popular as they do not involve rib spreading or mechanical retractors, which are seen in traditional thoracotomy. These techniques have also developed over the years to include robot-assisted lobectomy, uniportal resection, and awake VATS [9]. Several studies have shown that VATS lobectomy is associated with reduced postoperative pain and preserved postoperative pulmonary function. For example, a retrospective analysis of 1079 patients at Duke University found that VATS lobectomy was associated with lower postoperative complications, including a decrease in prolonged air leak, atrial fibrillation, atelectasis, pneumonia, renal failure, blood transfusions, and death compared to thoracotomies [10]. Recent studies have also revealed that the conversion rate from VATS to thoracotomy can reach as low as 3 to 5% [11,12], which is considerably less than in the studies in the past, with an approximately 11% conversion in one trial [13]. Additionally, it was found that patients who undergo VATS have a decreased cytokine release and lower levels of C-reactive protein, which may result in decreased rates of atelectasis and relatively preserved postoperative lung function [14,15]. 

Robotic-assisted thoracoscopic surgery (RATS) is another option, with OS rates of 91% for stage IA cancer and 88% for stage IB cancer [16]. A meta-analysis by Singer et al. [17] demonstrated that RATS lobectomy costs more than VATS lobectomy. A nationwide comparative study published in 2014 by Paul et al. obtained similar results while further identifying an association of robotic lobectomy with increased rates of intraoperative injury and bleeding [18]. On the contrary, numerous recent studies revealed no significant difference in outcomes, while others exhibited better outcomes with robotic surgery. A 2019 meta-analysis demonstrated no significant difference in the short-term outcomes between VATS and RATS [19]. Another study conducted by the Society of Thoracic Surgeons (STS) saw no significant difference in OS between the two approaches [20]. Additional studies found improved 30-day mortality, number of conversions to open surgery [21,22], decreased postoperative complications, and reduced hospital stay [23]. The ROMAN trial recently published its findings on the perioperative outcomes of RATS and VATS for early stage NSCLC. Although there were inconclusive findings favoring RATS, it revealed notably improved lymph node sampling with RATS [24]. Another trial conducted by Catelli et al. found no difference in OS and disease-free survival (DFS), and instead found lower postoperative complications, such as pleural effusion, pain, and cardiovascular comorbidities, using RATS [25]. A recent meta-analysis, which included 25 studies, including 5 randomized controlled trials (RCTs), compared the postoperative quality of life (QoL) of RATS and robotic abdominal surgery with VATS and laparoscopic surgery (LS). They found no significant difference in global QoL with the robotic techniques compared to VATS [26].

Uniportal lobectomy is a minimally invasive technique that involves operating through a single access incision, removing the need to create an additional camera port [27,28]. A large European multicentric retrospective cohort comparing uniportal with multiportal surgery discovered no significant difference in the number of lymph nodes extracted and the conversion rate to open surgery [29]. Conversely, they found a statistically significant lower operative time and decreased hospital stay in the uniportal group. A randomized control trial conducted by Yao et al. saw no difference in operative time, lymph nodes harvested, chest tube duration, length of hospital stay, and pulmonary function; however, intraoperative blood loss and volume of total drainage were significantly decreased with uniportal VATS [30]. Similar outcomes between uniportal VATS and other VAT techniques were compared by Perna et al. in their prospective, randomized study. They concluded that uniportal VATS does not yield superior outcomes compared to other techniques of VATS [31]. A meta-analysis conducted in 2019, comparing open surgery, uniportal VATS, multiportal VATS, and RATS obtained equivalent findings. An emerging technique that is gaining popularity is uniportal subxiphoid VATS, known for its potential to reduce pain by avoiding intercostal nerve injury [32]. An added benefit of this approach is the possibility of performing bilateral procedures without the need for extra incisions or time spent repositioning the patient. A retrospective cohort saw comparable results to other techniques with uniportal subxiphoid VATS [33]. Nevertheless, clinical trials are required to compare the outcomes of uniportal subxiphoid VATS with other minimally invasive techniques.

#### 3.1.2. Is Sublobar Resection Superior to Lobectomy for Stage Ia NSCLC Treatment?

While lobectomies have been the gold standard surgical resection in the treatment of early stage lung cancer since 1960, sublobar resection, comprising either segmentectomy or wedge resection, presents a notable difference in the surgical intensity. Segmentectomy is considered an alternative to lobectomy in terms of curative intensity in oncology, allowing for margin-positive or nodal metastasis to be assessed during surgery, while simultaneously being similar to wedge resection in terms of preservation of pulmonary parenchyma and postoperative respiratory function [34]. A recent clinical trial by Altorki et al. compared 362 individuals with peripheral cT1aN0 non-small cell lung cancer treated with either lobectomy, segmentectomy, or wedge resection. The outcomes they measured included DFS, OS, lung cancer-specific survival (LCSS), differences in surgical margins, locoregional recurrence rate, and expiratory flow rate at 6 months postoperatively. They found no significant difference in DFS, OS, LCSS, or pulmonary function between the three groups. Locoregional recurrence was numerically higher in wedge resection compared to segmentectomy but not statistically significant [35]. Another multicenter, noninferiority, phase 3 trial by Altorki and colleagues was conducted on a total of 697 patients with NSCLC clinically staged as T1aN0, who were randomly assigned to undergo sublobar resection or lobar resection after intraoperative confirmation of node-negative disease. The 5-year DFS rates after sublobar resection and lobectomy were 63.6% (95% CI, 57.9–68.8) and 64.1% (95% CI, 58.5–69.0), respectively. Hence, they concluded that sublobar resection was non-inferior to lobectomy in terms of DFS in patients with pathologically confirmed hilar and mediastinal lymph node-negative peripheral NSCLC [36]. A multicenter, open-label, phase 3 trial compared survival rates, mortality causes, and risk of recurrence between the two approaches in purely solid NSCLC less than or equal to 2cm. Their post hoc, supplemental analysis revealed a significantly improved 5-year OS with segmentectomy (86.1% [95% CI 81.4–89.7] with lobectomy vs. 92·4% [88.6-95.0] with segmentectomy). They further saw no statistically significant difference in the 5-year RFS (81.7% [95% CI 76·5–85·8] with lobectomy vs. 82.0% [76.9–86.0] with segmentectomy; HR 1.01 [95% CI 0.72–1.42]; *p* = 0.94). However, when considering demographics, better outcomes were observed with lobectomies in patients younger than 70 years (*p* = 0.049) and female patients (*p* = 0.047) [37]. Potter et al. aimed to compare these outcomes with the National Cancer Database in the United States in a propensity score-matched analysis. They found no significant difference in the 5-year OS between the two groups. Furthermore, subgroup analyses by histology and tumor grade exhibited no difference. Similar treatment patterns were also observed between the two approaches for second primary tumors [38]. A recent meta-analysis of randomized clinical trials comparing sublobar to lobar resection in stage IA NSCLC showed sublobar resection and lobectomy to have similar OS, DFS, and disease recurrence rates for stage IA NSCLC [39]. Another meta-analysis by Fong et al. also revealed similar outcomes, adding that sublobar resection ensures safer future treatments for patients experiencing recurrence or a second primary tumor [40]. A cross-sectional study by Brunelli et al. discussed dyspnea after segmentectomy versus lobectomy, comparing their Dyspnea Index Score. They found a reduced chance of perioperative dyspnea in the segmentectomy group. 

These recent studies indicate that sublobar resection is a feasible alternative to lobectomy in NSCLC management. Numerous outcomes, including DFS, OS, and LCSS, show no significant difference between the two, particularly between segmentectomy and lobectomy. However, the data in terms of preservation of pulmonary function between sublobar resection and lobectomy also remain inconclusive. Therefore, more clinical trials may be required to determine any significant differences between the outcomes of these options. 

#### 3.1.3. What Is the Prognostic Value of Ground-Glass Opacities (GGOs)?

Ground-glass opacity (GGO) is defined as an area of hazy attenuation on CT scans with visible underlying blood vessels and bronchial structures [41]. GGOs are typically associated with adenocarcinomas, although they may be present in certain pulmonary conditions, such as COVID-19, potentially posing diagnostic challenges as the GGOs from such benign conditions mimic the ones observed in malignancy [42]. New imaging technologies are necessary to identify neoplastic or potential neoplastic GGOs which need operation. Pulmonary nodules possessing a GGO component are known as subsolid nodules (SSNs). SSNs are further divided into pure GGOs and part-solid GGOs [43]. The degree of GGO is measured using the consolidation-to-tumor ratio (CTR), defined as the solid portion size relative to the total size of the nodule [44]. The degree of malignancy has been associated with the proportion of GGO in each nodule, with the literature showing that nodules with large GGO components have a favorable prognosis [44,45,46,47,48]. Shigefuku et al. noted a positive impact of GGO on recurrence and 5-year survival after resection of adenocarcinoma [49]. Multifocal pure GGOs have exhibited a significantly higher 5-year OS (97.2%) compared to having a purely solid nodule (PSN) with additional GGOs (82.1%) or having only PSNs (41.3%) [50]. A recent cohort study by Choi et al. compared the metastatic potential of GGOs and PSNs with an increase in tumor size. Tumor size was observed as a significant predictor of outcomes in a multivariate analysis for the PSN, but not the GGO group. The GGO group also had a superior 5-year DFS [51]. Hence, while some studies found no association between CTR and tumor prognosis [52,53], the majority suggested the utilization of CTR to assess the T stage [40,45,54]. This prognosis may also differ based on the histologic characteristics of SSNs (Table 1) [40,55]. 

Due to their favorable prognostic value, possible alternative options to surgery for patients with GGOs have also been explored, particularly in patients who may be inoperable due to comorbidities, present with multiple lesions, or refuse surgery. Stereotactic body radiotherapy (SBRT) is one such option, proving to be a safe monotherapy with low toxicity for SSNs with a CTR ≤ 0.5 in a recent study [56]. Notably, in a retrospective study by Eriguchi et al., SBRT achieved a 3-year OS and cause-specific survival (CSS) of 100% for GGO tumors in operable patients [57]. Another study observed similar findings, with 3-year RFS and CSS rates of 96.0% and 100.0%, respectively. Furthermore, they noted no significant difference in the 3-year OS and RFS between operable and inoperable patients. Both these studies, therefore, explored the possibility of using stereotactic radiotherapy even in individuals who are deemed suitable for surgery. Carbon ion radiotherapy (CIRT) is another alternative, with one study revealing a Kaplan–Meier estimate of OS being significantly lower after CIRT than segmentectomy but with similar CSS [58]. Additionally, percutaneous radiofrequency ablation (RFA) could be used, with one study observing an OS and CSS of 96.4% and 100% at 3 years, and 96.4% and 100% at 5 years, respectively [59]. Lastly, another study by Iguchi et al. utilizing RFA found an OS and CSS of 93.3% and 100%, respectively, at 1 and 5 years [60]. Comparing the QoL of segmentectomy with SBRT has also been studied using the Short Form 8 (SF-8), for physical and mental health, and Functional Assessment of Cancer Therapy-Lung (FACT-L) surveys [61]. Patients reported better QoL immediately postop with SBRT but no significant difference between the two in long-term QoL. It is important to note that these studies are retrospective, with some having a small sample size; hence, a further evaluation with clinical trials is recommended before they can be routinely utilized for GGO management.

#### 3.1.4. Is Sublobar Resection Superior to Lobectomy for GGOs?

The prevalence of GGOs has risen due to early detection from the application of lung cancer screening and CT scans. More GGOs are now being recognized in their early stages, thus increasing the feasibility of sublobar resection, such as wedge resection and segmentectomies, compared to lobectomies. A recent large cohort study included 1209 patients who either underwent wedge resection or segmentectomy. Wedge resection was found to have a significantly lower complication rate, shorter operating time, and shorter hospital stay. Along with that, they discovered statistically similar 5-year OS (98.8% vs. 99.6%, *p* = 0.270), 5-year RFS (98.8% vs. 99.5%, *p* = 0.307), and 5-year LCSS (99.9% vs. 99.6%, *p* = 0.581) with wedge resection and segmentectomy, respectively [62]. Another retrospective cohort by Zhang et al. included 424 patients with part-solid GGOs. They also discovered improved operative time, blood loss, and postoperative stay with sublobar resection. In addition, they saw similar postoperative complications and OS between the two for GGO-dominant lung adenocarcinomas ≤ 2 cm [63]. The Japan Clinical Oncology Group (JCOG) 1211 trial, a multicenter, single-arm, confirmatory phase 3 trial, confirmed these findings [64]. There is a need, however, for more clinical trials to better validate these findings. The ongoing GREAT trial is a prospective, open-label, randomized phase III trial across 19 hospitals in China, randomizing 1024 patients into segmentectomy and lobectomy. Their primary endpoint is 5-year RFS, and secondary endpoints include 5-year OS, perioperative outcomes, and pulmonary function preservation. They expect improved secondary endpoints and no statistical difference in the primary endpoint [65].

#### 3.1.5. What Is the Extent of Lymphadenectomy Required for GGOs?

Lymphadenectomy, which includes lymph node sampling (LNS), and the more extensive lymph node dissection (LND), is an important component of NSCLC management. Due to the rise in the detection of early stage GGOs, the clinical significance of LND needs to be evaluated. A recent retrospective cohort study aimed to analyze the difference in clinical outcomes between LND and sampling for a CTR between 0.3 and 0.7. The Kaplan–Meier survival curves found similar outcomes for both approaches [66]. Another recent cohort concluded that complete exclusion of lymphadenectomy has a minimal impact on the curative management of GGOs for both sublobar and lobar resection [67]. A review by Kim et al. included numerous studies, including five clinical trials, discussing the extent of lymphadenectomy [68]. They discovered no significant difference in postoperative morbidities between lymph node sampling and dissection, with two studies noting an improved detection of occult N2 disease with dissection, and two other studies showing improved survival after dissection. However, they also noted methodologic uncertainties and a high risk of bias for all studies [68]. This was further highlighted in a meta-analysis of these studies. They saw a favorable OS but more complications with dissection. Nonetheless, they alluded to the limitations of the studies, particularly mentioning the asserted survival advantage not being backed up with reliable evidence [69]. Both reviews emphasized the need for larger randomized clinical trials that are more regulated. Another review by Deng et al. added that the studies they evaluated did not prove a survival benefit with dissection [70]. Moreover, five retrospective studies they referred to reported no or minimal lymph node involvement with pure GGOs and part-solid GGOs, respectively. With this, they suggested that lymph node dissection may not be required for pure GGOs and some part-solid GGOs. In contrast to the preceding two reviews, they also acknowledged that considering this excellent prognosis of GGOs, along with the intricacy of conducting RCTs, which demand excessive sampling and follow-up time, RCTs may not be imperative to determine the optimal lymphadenectomy strategy for GGOs, although studies are needed to understand lymphadenectomy for NSCLC in general [71]. Currently, two ongoing trials are assessing approaches to lymph node removal in GGOs. The LESSON trial is an ongoing, single-institutional, randomized, double-blind, and parallel-controlled trial in China aiming to assess lymph node dissection in clinically diagnosed stage IA NSCLC with GGO components ≥50% (i.e., CTR ≤ 0.5) [71]. The MELDSIG trial is another ongoing multi-institutional randomized trial in China, analyzing the difference between dissection and sampling in stage Ia NSCLC with GGOs [72].

### 3.2. Non-Surgical Approaches to NSCLC

#### 3.2.1. Radiotherapy and Adjuvant Chemotherapy

Patients who are medically unable to undergo surgery for early stage NSCLC are usually treated with radical radiotherapy. However, when standard fractionation is used, the outcomes are not as good as surgery, with 5-year OS rates of only 11% [73]. On the other hand, using stereotactic ablative radiotherapy (SABR) has shown similar local control rates and disease-specific survival rates to surgery [74]. Adjuvant cisplatin-based doublet chemotherapy has become the standard of care for completely resected stage II NSCLC based on the International Adjuvant Lung Cancer Trial in 2004 [75], but no significant innovations have been made since then. A phase II randomized TREAT study evaluated the role of cisplatin-pemetrexed versus cisplatin-vinorelbine, but a follow-up report showed no improvement in the 3-year survival period [76]. The addition of bevacizumab and erlotinib did not improve survival in the Eastern Cooperative Oncology Group 1505 study [77] nor the RADIANT study [78], respectively.

#### 3.2.2. Immunotherapy

The mainstay for treating early stage NSCLC has traditionally been surgery alone. However, adjuvant immunotherapy has been proposed to reduce recurrence and facilitate cancer destruction. Surgery can cause immune dysfunction [79], which may allow unresected cancer cells to grow, but the use of adjuvant immunotherapy allows the timely treatment of subclinical micrometastatic disease [80]. Due to the groundbreaking outcomes of immune checkpoint inhibition (ICI) for metastatic (stage IV) NSCLC [81,82,83], investigating its potential in early stage NSCLC made sense. In addition, the success of durvalumab ICI in treating stage III unresectable NSCLC has increased interest in using ICI for non-metastatic early stage NSCLC [84].

Currently, four large randomized controlled phase III trials are investigating the use of ICI as an adjuvant treatment after surgical resection. These trials include PEARLS [85], Canadian Cancer Trials Group BR.31 [86], ANVIL [87], and IMpower010 [88]. All trials are conducted on patients with completely resected stage IB more than 4 cm, II, or IIIA, and allow adjuvant chemotherapy as per standard practice. Most allow resected tumors of any programmed death ligand 1(PD-L1) status, but the BR.31 trial will enrich the trial population with PD-L1-positive tumors after the enrollment of 600 patients. Two trials are placebo-controlled, whereas the ANVIL and IMpower010 are not. DFS is the primary endpoint in PEARLS. For the BR.31, this is DFS in PD-L1-positive tumors. IMpower010 has both endpoints, and ANVIL targets DFS and OS.

The IMpower010 trial showed a DFS benefit with atezolizumab, a PD-L1 inhibitor, versus best supportive care after adjuvant chemotherapy in patients with resected early stage NSCLC, with a pronounced benefit in the subgroup whose tumors expressed PD-L1 on 1% or more of tumor cells, and no new safety signals. However, there are certain disadvantages to neoadjuvant immunotherapy. First, it is unclear whether it will improve the patient’s long-term survival. Second, it may jeopardize surgical feasibility by generating delays or raising the risk of complications. Furthermore, there are challenges in measuring the response and investigating biomarkers, which may limit its applicability and advancement.

#### 3.2.3. Targeted Therapy

Targeted therapy using tyrosine kinase inhibitors (TKIs) has shown promise as an adjuvant treatment for *EGFR*-mutated NSCLC. The SELECT trial [89] found that adjuvant erlotinib improved 2-year DFS compared to historical controls. The CTONG1104/ADJUVANT trial [90] compared standard chemotherapy to gefitinib and found a superior DFS in the gefitinib arm. The ADAURA trial evaluated the impact of adjuvant osimertinib compared to a placebo and found an impressive DFS hazard ratio of 0.17 (95% CI 0.12–0.23, *p* < 0.05); however, controversy remains about whether these immature data should change practice. Additionally, neoadjuvant gefitinib has shown a 50% response rate among patients whose tumors harbored EGFR mutations, without a safety signal for increased surgical risk [91]. MET is a tyrosine kinase receptor for hepatocyte growth factor. *MET* gene amplification is observed in 2 to 4 percent of treatment-naïve NSCLC and in 5 to 20 percent of *EGFR*-mutated tumors that have acquired resistance to EGFR inhibitors. The literature suggests the use of MET inhibitors, such as capmatinib or crizotinib, in patients with a high-level MET amplification (>5-fold increase in gene copy number [GCN] or MET/CEP7 ratio >5) who have progressed despite being on chemotherapy or immunotherapy [92]. Hyperactivation mutations of the PI3K–AKT–mTOR signaling pathway are observed in many cancers, including NSCLC, where they have been heavily implicated in carcinogenesis and disease progression. Pilaralisib is a highly selective inhibitor of the class I PI3Ks and successfully inhibits tumor growth in vivo. Crizotinib is an ALK, MET, and ROS1 kinase inhibitor. The phase I study of Crizotinib in 50 patients who were positive for ROS1 rearrangement proved the antitumor activity of this drug in advanced NSCLC [93]. 

## 4. Future Directions and Conclusions

More research on early stage non-small cell lung cancer (NSCLC) is crucial to improve outcomes and find more effective treatments. With the increasing prevalence of early stage NSCLC, surgical techniques involving minimal resection of the lung parenchyma, i.e., sublobar resections, need to be explored, aiming to preserve function and minimize operative and postoperative complications. Moreover, while the prognostic significance of SSNs with major GGO components has been extensively studied, there is limited data regarding the relevance of GGOs with a CTR > 0.5. Studying the clinical progression of SSNs, such as lymph node involvement, will allow for the development of better treatment protocols, including the extent of lung resection, the extent of lymphadenectomy, and the utilization of non-surgical approaches. Lastly, as the range of treatment options expands, there is an increasing demand for a customized approach that incorporates a combination of surgical and non-surgical therapies and personalized medicine [94]. Potential selection biases in the reviewed studies, often from high-income countries, may limit generalizability. Additionally, many studies on surgical and non-surgical treatments like radiotherapy and ablation are retrospective, which can introduce biases, affecting their conclusions. More clinical trials are needed, and they are needed for a variety of populations to provide more generalizable conclusions.

## Figures and Tables

**Table 1:** Lepidic tumors presenting with GGO components.

Histologic Type	Size	Description
Atypical adenomatous hyperplasia (AAH)	Usually ≤0.5 cm	Solitary GGN usually smaller than 0.5 cm with no solid components
Adenocarcinoma in situ (AIS)	≤3 cm	Solitary GGN with purely lepidic growth, no stromal components, vascular, pleural and lymphatics invasion, or necrosis
Minimally invasive adenocarcinoma (MIA)	≤3 cm	Solitary GGN with mainly lepidic growth, ≤0.5 cm invasive foci, no stromal components, vascular, pleural, and lymphatics invasion, or necrosis
Lepidic predominant adenocarcinoma (LPA)	Any total size	Mainly lepidic growth, >0.5 cm invasive foci, or vascular, pleural, and lymphatics invasion, or necrosis
